# Noise Normalizes Firing Output of Mouse Lateral Geniculate Nucleus Neurons

**DOI:** 10.1371/journal.pone.0057961

**Published:** 2013-02-28

**Authors:** Rajiv Wijesinghe, Samuel G. Solomon, Aaron J. Camp

**Affiliations:** Sydney Medical School, School of Medical Sciences and Bosch Institute, The University of Sydney, New South Wales, Australia; Tokai University, Japan

## Abstract

The output of individual neurons is dependent on both synaptic and intrinsic membrane properties. While it is clear that the response of an individual neuron can be facilitated or inhibited based on the summation of its constituent synaptic inputs, it is not clear whether subthreshold activity, (e.g. synaptic “noise”- fluctuations that do not change the mean membrane potential) also serve a function in the control of neuronal output. Here we studied this by making whole-cell patch-clamp recordings from 29 mouse thalamocortical relay (TC) neurons. For each neuron we measured neuronal gain in response to either injection of current noise, or activation of the metabotropic glutamate receptor-mediated cortical feedback network (synaptic noise). As expected, injection of current noise via the recording pipette induces shifts in neuronal gain that are dependent on the amplitude of current noise, such that larger shifts in gain are observed in response to larger amplitude noise injections. Importantly we show that shifts in neuronal gain are also dependent on the intrinsic sensitivity of the neuron tested, such that the gain of intrinsically sensitive neurons is attenuated divisively in response to current noise, while the gain of insensitive neurons is facilitated multiplicatively by injection of current noise- effectively normalizing the output of the dLGN as a whole. In contrast, when the cortical feedback network was activated, only multiplicative gain changes were observed. These network activation-dependent changes were associated with reductions in the slow afterhyperpolarization (sAHP), and were mediated at least in part, by T-type calcium channels. Together, this suggests that TC neurons have the machinery necessary to compute multiple output solutions to a given set of stimuli depending on the current level of network stimulation.

## Introduction

Individual thalamocortical relay (TC) neurons can mediate non-linear signal transformations, which may be important for both the gating and information processing functions of the thalamus. For example, the expression of low-voltage activated T-type Ca^2+^ channels [1], [2], [3], important in the generation of brain rhythms [4], [5], [6], confers two distinct response modes on TC neurons [7], [8], [9]. The mode of firing – ‘burst’ or ‘tonic’ – depends upon the recent membrane potential history [9], [10], which can be modulated by synaptic inputs [11], [12]. In particular, the sign of retinal (feedforward) inputs onto TC neurons determines which mode of firing is recruited to signal specific features of a visual scene [13], [14], [15], [16]. These studies show that individual TC neurons have the cellular machinery necessary to provide adaptive computations over their inputs.

The lateral geniculate nucleus (LGN), the primary relay of retinal signals to the visual cortex, has proved to be a useful model system for studying thalamic function [17]. Anatomical studies demonstrate that TC neurons receive a wide range of inputs from cortical, subcortical, and peripheral sensory structures [18], [19], [20]. Many of these have addressed the peculiar advantages of the ‘burst’ firing mode [21], [22], but during normal processing it is the tonic-firing mode that predominates, providing over 90% of spikes. Here we asked whether the output of TC neurons during both discharge patterns were affected by specific network activation states. Specifically, we investigated the mechanisms by which TC neurons adjust their sensitivity (firing threshold and gain) to simulated network activity (via injected current noise) and physiologically relevant activity (via the metabotropic glutamate receptor-mediated corticothalamic feedback pathway- which accounts for 30% of inputs to these neurons [23]). Studies in rat somatosensory cortex and guinea pig thalamus have shown that increasing the amount of current noise reduces the gain of neurons [24], [25], while studies in somatosensory cortex [26] have shown that different types of neurons may respond differently to noise. By conducting patch-clamp recordings from mouse dLGN TC neurons, we show that simulated network activity (current-noise) and physiological activity (excitatory corticothalamic feedback) increase gain on average. In addition, simulated network activity also reduced gain in a minority of neurons, suggesting that the prevailing level of network activation may perform a normalisation operation, tending to set the sensitivity of neurons at an optimal value.

## Methods

### Ethics Statement

All procedures were approved by the Animal Care and Ethics Committee of the University of Sydney (protocol number K22/6-2009/3/5042).

### Animals and Tissue Preparation

All experiments were performed on juvenile (22–60 days) male C57BL/6 mice [27]. All chemicals were obtained from Sigma Aldrich (Castle Hill, Australia) unless otherwise specified. Mice were deeply anaesthetized with an intraperitoneal injection of Ketamine (1 mg/kg; Parnell, Alexandria, Australia) and decapitated. The parietal and occipital bones were removed to expose the dorsal region of the brain. During this procedure the brain was constantly bathed in ice-cold sucrose-based artificial cerebrospinal fluid (sACSF) that contained (in mM): 236 sucrose, 26 NaHCO_3_, 11 glucose, 3 KCl, 1.25 NaH_2_PO_4_, 2 MgCl_2_, 2.5 CaCl_2_. This solution was continually gassed with Carbogen (95% O_2_, 5% CO_2_) to achieve a final pH of 7.2–7.3 [27], [28]. To isolate the dLGN, two cuts were made in the coronal plane; one approximately 1 mm rostral and the other approximately 4 mm caudal to bregma. This block of tissue was removed from the skull and secured caudal face down to the stage of a vibrating microtome (DSK Microslicer DTK-1000, Kyoto, Japan) using cyanoacrylate glue (Selleys, Padstow, Australia). This setup was transferred to a cutting chamber filled with ice-cold, continually oxygenized sACSF. Coronal slices (250 µm thick) were cut, and those containing the LGN (3–4 slices [29]) were transferred to an incubation chamber containing ACSF (120 mM NaCl substituted for sucrose), at room temperature (21°C) and allowed to equilibrate for 1.5 hrs prior to recording.

### Electrophysiology

**Figure 1:**
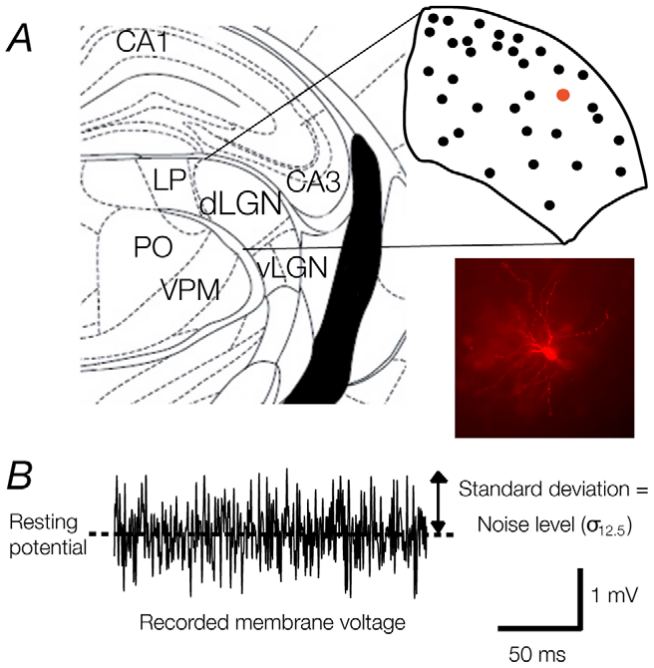
Schematic of the mouse dorsal lateral geniculate nucleus (dLGN) and representative noise stimuli. **A**. The dLGN is shown in relation the hippocampus (CA3 and CA1), ventral lateral geniculate nucleus (vLGN), lateral posterior nucleus (LP), posterior nucleus (PO), and the medial portion of the posterior nucleus (VPM) in a coronal plane (2.06 mm caudal to Bregma, left hemisphere). Inset shows the map of recording sites within the dLGN. Note that cells were recorded throughout the dorsoventral, and mediolateral extent of the LGN (Plates 45–51 in Paxinos and Watson, 2008. On the bottom right is a photo of a representative TC neuron. **B**. The response of a cell to a noisy current stimulus with a mean current of 0 pA. The value σ_12.5_ for current noise of different standard deviations (n) was calculated as the standard deviation of the recorded membrane potential. The noise levels presented throughout are the average of the standard deviation (caused by this stimulus for each n) across all cells tested.

After incubation slices were transferred to a small glass-bottom recording chamber and secured by a weighted nylon net. The chamber was continually perfused (5–6 bath volumes/min) with oxygenized ACSF at 32 ± 1°C. Slices were viewed using a fixed-stage microscope (Olympus BX-51WI, Tokyo, Japan) at low power (10x) to identify the dLGN. Thalamic neurons were visually identified using near infra-red differential interference contrast optics and a high power (40x) water-immersion lens. Micropipettes were pulled from thin-walled borosilicate glass tubing (1.5 mm OD, Warner Instruments, Hamden, Connecticut) using a micropipette puller (Narishige, Tokyo, Japan). Pipettes were filled with a potassium-based internal electrode solution containing (in mM): 70 potassium gluconate, 70 KCl, 2 NaCl, 10 HEPES, 4 EGTA, 4 Mg_2_-ATP, 0.3 Na_3_-GTP. The pH was adjusted using KOH to give a final pH of 7.3 and an osmolarity of 290 mOsmol [27], [30]. Lucifer yellow (0.5 mg/mL, Invitrogen, Eugene, Oregon) was included in the internal solution to allow for post-recording morphological analysis of individual neurons and mapping of recording sites. Recording pipettes (final resistance of 4–7 MΩ) were positioned in the recording chamber using a motorised micromanipulator (Sutter, Nuslock City, Germany). Voltage data was corrected for a measured junction potential of -6 mV, and fast and slow capacitance was uncompensated. Targeted recordings were made throughout the anatomical extent of the dLGN to sample from the largest possible cell population (Figure 1).

Whole-cell current clamp recordings were made using a Multiclamp 700B amplifier (Molecular Devices, Sunnyvale, California, USA). Records were sampled at 10 kHz using an ITC-18 digitiser (Instrutech, California). Data acquisition was performed on an Intel-based Apple Macintosh iMac computer using Axograph X v1.3 acquisition software (Axograph Scientific, Sydney, Australia). Analysis was carried out using software packages within Axograph X or Igor Pro 6.01 (Wavemetrics, Lake Oswego, OR, USA). Recordings were made from cells with resting membrane potentials between -60 and -75 mV and input resistances greater than 40 MΩ, a criterion satisfied by all but 4 of 33 cells targeted. During whole-cell current clamp recordings, the following drugs were added to the ACSF as required: 250 µM NiSO_4_ (Ni^2+^, T-type Ca^2+^ channel blocker), 100 nM tetrodotoxin (TTX), 250 mM 1-aminocyclopentane-trans-1,3-dicarboxylic acid (trans-ACPD; mGluR1a agonist, Tocris Bioscience, Brisbane, Australia).

### Immunocytochemistry

Slices from which successful recordings were made were fixed in 4% paraformaldehyde for 24 h post-recording. Slices were then washed in 0.1 M phosphate-buffered saline (PBS) for 1 h, pre-incubated in a solution of 5% bovine serum albumin (BSA) and 0.5% Triton x100 in 0.1 M PBS for 1 h, and incubated in a primary antibody solution containing 1% BSA, 0.5% Triton x100, 0.1% sodium azide and rabbit anti-Lucifer yellow IgG (2 mg/uL, Invitrogen, Mulgrave, Australia) in 0.1 M PBS for 5 days. Slices were then washed in PBS overnight and incubated in a secondary antibody solution containing 1% BSA, 0.25% Triton x100, 0.1% sodium azide and goat-anti rabbit IgG conjugated with Alexa594 (2 mg/uL, Invitrogen) in 0.1 M PBS overnight. Slices were then washed in PBS and mounted onto glass slides using Citifluor anti-fadent mounting media (Proscitech, Kirwan, Australia) and a plastic coverslip. Labelled cells were imaged using a Zeiss Axiovert microscope (Oberkochen, Germany) equipped with a 40x oil-immersion lens and a 585 nm emission filter.

### Current stimuli

‘Noisy’ current steps were 1 second in duration and generated at the rate at which data was acquired (10 kHz). The magnitude of the current at each time point was randomly drawn from a Gaussian distribution (zero-mean) to create a sample of ‘white’ noise, which was subsequently low-pass filtered at 2 kHz. The amplitude of this sample was scaled so that the standard deviation was 6.25, 12.5, 25 or 50 pA. The noise was injected alone, or was added to a step function of the same duration, the amplitude of which ranged from 100 pA to 200 pA (10 pA increments). Responses were obtained to each of the 4 noise amplitudes, at each of the step amplitudes. To represent the injected noise in a relevant way the noise level (σ_n_, where n is the standard deviation of the injected current) that we show is the standard deviation of the membrane potential during injection of the noise stimulus alone.

### Data Analysis

Passive membrane properties, including impedance and capacitance, were measured before and after the quantitative measurements described here, and were derived from the response to 10 ms, 5 mV pulse delivered in voltage-clamp mode from a holding potential of −70 mV. Data were rejected if these parameters changed by more than 20% during the course of an experiment.

#### Discharge Properties

Resting membrane potential was calculated as the average potential during the 500 ms preceding each stimulus. Only spikes with amplitudes that exceeded a threshold value of 0 mV (overshooting spikes) were included for analysis. The stimulus-afterhyperpolarisation (sAHP) was defined as the difference between the minimum membrane potential after stimulus offset and the resting membrane potential. Pairs of spikes with inter-spike time intervals (i.s.i’s) of < 5 ms were classified as ‘burst spikes’, while the remainder were classified as ‘tonic spikes’. Spike frequency was calculated by counting the number of spikes occurring over the 1-second depolarizing current step. Frequency was then plotted against the magnitude of injected current (f-I plot) from the responses to a set current steps (20 pA increments, 0 to 400 pA) to calculate two measures of sensitivity: 1) threshold, defined as the minimum input strength necessary to evoke discharge tonic discharge > 3 Hz, and 2) gain, calculated as the slope of the linear regression for responses above threshold. To ensure that measures of sensitivity were not masked by the size of the recorded cell, threshold and gain estimates were normalised by multiplying each parameter by the inverse of the recorded impedance of each cell.

#### Quantifying influence of simulated inputs

The variance in membrane potential was used to assess the impact of trans-ACPD in activating synaptic inputs on TC cells. The noise level (σ_n_) was defined as the standard deviation of the membrane potential in response to a noise stimulus delivered in the absence of an increase in mean current. For control estimates, the variance was calculated over a 30 second window before the application of the trans-ACPD, and for drug-induced values during a 30 second window 5 minutes after the beginning of drug application.

#### Statistical analysis

Student’s *t*-tests were used for comparisons between variables under control and drug application, or in the presence of noise. When analysing the distributions of threshold and gain we used a Kolomogorov-Smirnov test for normality. Significance was set at p < 0.05. All errors are presented as the standard error of the mean (SEM) unless otherwise stated.

## Results

Whole-cell current clamp recordings were obtained from 29 identified dLGN neurons from 15 animals, as part of a larger set of experiments. Visual criteria, including large soma size and degree of dendritic arborization, were used to target putative thalamocortical (TC) relay neurons. Electrophysiological criteria, including the presence of low-threshold action potentials and a depolarising sag in response to hyperpolarising pulses [31], provided the TC classification. One neuron produced action potentials at a very low rate (<5 Hz) throughout the experiment and was excluded from analysis. The population of TC cells had the following passive membrane properties (mean ± SD): average resting membrane potential  =  -67 ± 4 mV, and input resistance  =  84 ± 5 MΩ. No cells produced action potentials spontaneously from resting membrane potential. Subsequent reconstruction of the recorded neurons was enabled by addition of lucifer-yellow to the recording pipette (Figure 1). The morphology of all the neurons included here was consistent with the known morphology of TC cells [32].

### Burst and tonic firing occurs from resting potential

**Figure 2:**
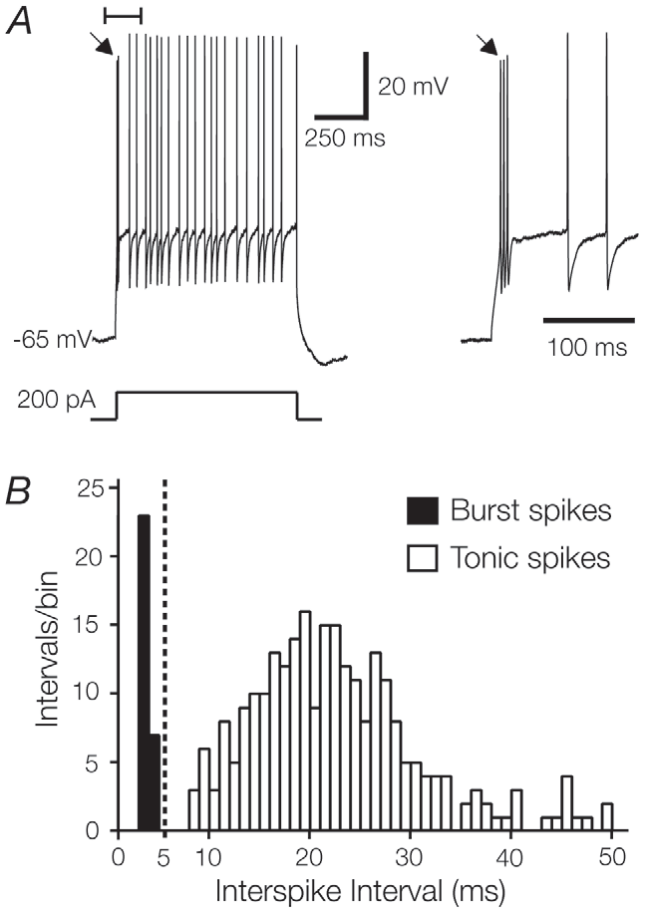
Burst and tonic spikes occur within exclusive temporal domains. **A**. The stereotypical response of a TC cell to a 1s, 200 pA depolarising current pulse delivered from resting membrane potential (-65 mV). The onset of the response (first 150 ms, inset) is characterised by a high frequency burst of spikes (246 Hz, arrow) followed by a shift to tonic firing. **B** . Interspike interval (i.s.i.) histogram (1 ms bin width) from a single TC cell in the response to a set of 20 current steps from 0 to 400 pA. Note the clear segregation either side of the 5 ms interval. Adjacent spikes with intervals shorter than 5 ms were classified as “burst” spikes, while those greater than 5 ms were classified as “tonic” spikes. Intervals greater than 50 ms (4 out of 693 in this example) were excluded from the plot for clarity.

At resting membrane potential all TC cells were silent. In response to a current pulse delivered from rest, TC cells produced action potentials in a stereotypical pattern. Figure 2A shows the typical response of a TC cell to a 1 s current pulse of 200 pA. At the beginning of the current step the cell responded with a burst of high frequency spikes (range: 2–7 spikes/burst, mode: 3 spikes/burst, n  =  28). The magnitude of this burst was independent of injected current; a current pulse sufficient to bring about a LTS led to a burst of spikes that was stable for each neuron. This burst of firing was followed by tonic firing in 24 of the 28 cells, which persisted throughout the current step, and followed the burst by a short latency (65.0 ± 18 ms, n  =  24). Unlike the initial burst response, the frequency of discharge in the tonic period increased with current amplitude (max: 117 Hz), but never approached the rate during burst firing (by definition >200 Hz).

The distinction between the burst and tonic firing modes is made clear by constructing a histogram of the interspike intervals (ISI’s) for every pair of spikes recorded during presentation of a set of 20 current steps (from 0 to 400 pA; Fig. 2B). The distribution segregated into two distinct groups; one containing all pairs of spikes with ISI ≤ 5 ms (black bars, Fig. 2B), the other containing pairs with ISI > 5 ms (white bars, Fig. 2B). All pairs of spikes with ISI ≤ 5 ms occurred within the ‘burst’ at the onset of each step, and all pairs of spikes during the tonic period showed ISI > 5 ms, regardless of the input current amplitude. This is consistent with the presence of two distinct firing modes in TC cells that occupy exclusive temporal domains when stimulated from resting membrane potential.

### Variability in the sensitivity of TC cells

**Figure 3:**
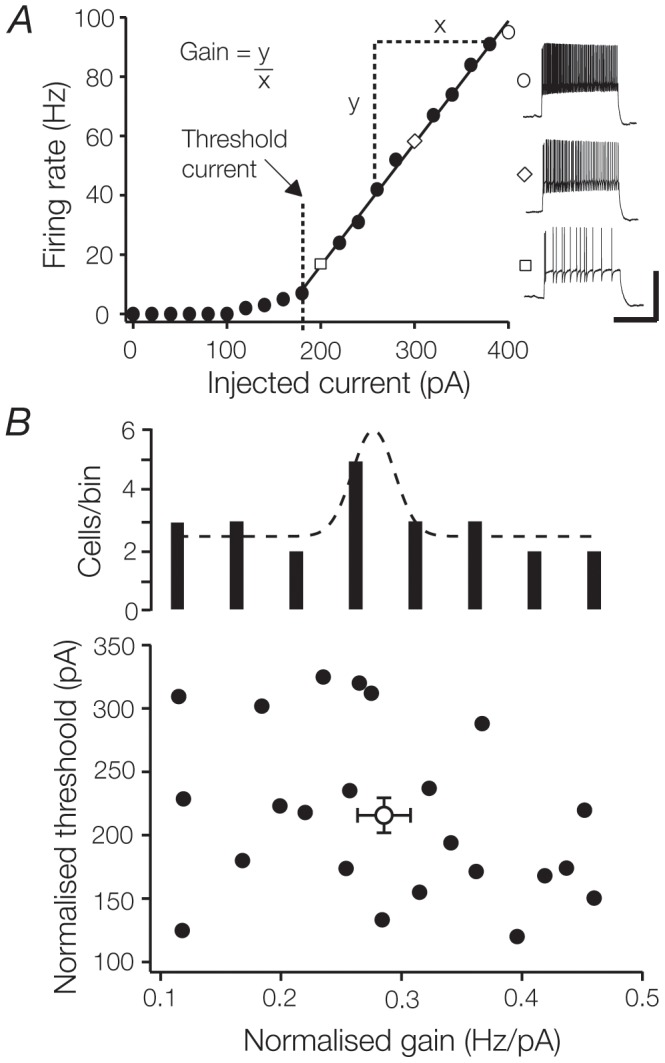
TC cells display a wide range of gains and thresholds. **A**. Firing rate as a function of input current amplitude (f-I relationship) for a typical TC cell. A straight line was fitted from the first point above tonic firing threshold to the last recorded response; the slope of this fit was a measure of gain. The gain and threshold of this neuron were 0.432 Hz/pA and 180 pA respectively. Shown to the right are representative traces recorded in response to 200, 300 and 400 pA (square, diamond, and circle respectively) current pulses. **B**. Firing threshold plotted as a function of gain. Both measures were normalised against the input resistance of each cell to minimize error associated with cell soma area. The average of each measure (and their respective SEMs) is indicated by the empty circle. A histogram of normalised gain (above) demonstrates that gains are normally distributed.

To analyse changes in the sensitivity of TC cells, we first needed to establish the baseline measures of gain and threshold in the absence of external influences**.** In the following we restrict our analysis to the tonic component of TC cell spiking activity. We do this because burst spiking provides no graded input-output relationship from which to infer sensitivity, and because the tonic mode represents a more dynamic component of the TC cell firing output. Figure 3A shows the spike frequency vs. current (f-I) relationship in response to current steps for a typical TC cell. In this and all cells, tonic discharge rates rose rapidly and relatively linearly following a threshold, before saturating at a discharge rate of near 100 Hz. The average tonic firing threshold (211 ± 15 pA, n  =  24) was 53% larger than the threshold for burst firing (143 ± 16 pA, n  =  24). We define the tonic threshold as the first current value capable of driving tonic discharge above 3 Hz, and calculated the gain as the slope of a linear fit to the straightest portion of the f-I plot. To allow comparison between cells, the estimated gain and threshold was normalised against input resistance (see Methods). Figure 3B plots these normalised values for the sample of TC cells. Gain was well described by a normal distribution (0.290 ± 0.02 Hz/pA, n  =  24, Fig. 3B; p < 0.01, one-sample Kolmogorov-Smirnov test), while thresholds appeared uniformly distributed. The input sensitivity of TC cells is therefore heterogeneous.

### Impact of current noise on input sensitivity

**Figure 4:**
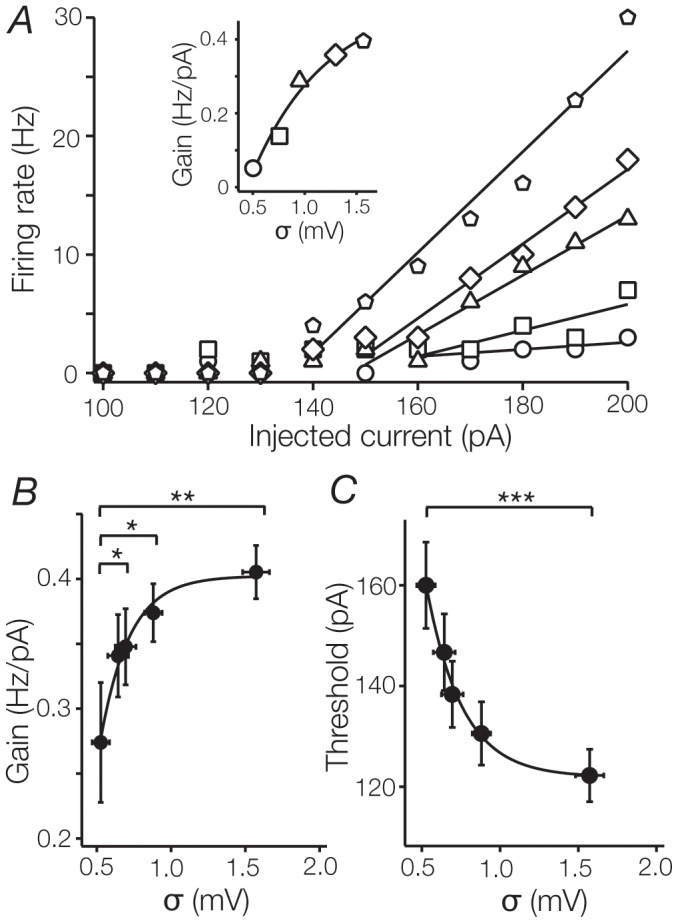
Noise induces both additive and multiplicative gain changes **A**. Shows for a typical TC cell the f-I relationships plotted at different levels of current noise (σ_n_, where σ is the standard deviation of the membrane potential in response to a ‘noisy’ current pulse with a mean current of 0 pA, and n represents the standard deviation of the injected current noise). In this example, the highest level of noise significantly increased the gain (0.05 to 0.39 Hz/pA; multiplicative gain change, indicated by an increase in the slope) and decreased the threshold (160 to 140 pA; additive gain change, indicated by a shift to the left) of this cell in comparison to control conditions. **B**. Gains averaged across the sample population plotted against noise level. On average, increasing levels of noise increased the gain of TC cells. Data points were well fit by an inverse exponential function, indicating that increases in gain saturate at high noise levels. **C**. Increasing levels of noise reduced the threshold of TC cells. As in B, this reduction saturated at high noise levels (between 1.0 and 1.5).

The output of individual cells is the eventual product of transformations imposed by synaptic inputs on intrinsic membrane properties. As a simple substitute for network activity, we asked how adding noise to the current pulse, simulating the addition of a background synaptic barrage, altered the gain of TC cells. Figure 4A shows the f-I relationship for a single TC cell in response to different levels of current noise. In this example, gain increased with increasing levels of noise (0.05 Hz/pA at σ_0_  =  0.05 mV, 0.39 Hz/pA at highest noise level, σ_50_  =  1.57 mV, Fig. 4A inset) and threshold decreased (σ_0_: 170 pA, σ_50_: 140 pA). Across our sample of cells the addition of noise increased gain from 0.27 ± 0.04 Hz/pA at σ_0_ =  0.53 ± 0.05 mV, to 0.41 ± 0.02 Hz/pA at σ_50_  =  1.57 ± 0.09 mV (n  =  18, p < 0.01, Fig. 4B). The increase in gain is consistent with a multiplicative transformation of neuronal output. Meanwhile, threshold decreased on average, from 160 ± 8.6 pA at σ_0_, to 122 ± 5.2 pA at σ_50_ (n  =  18, p < 0.001, Fig. 4C). This represents a leftward shift of the f-I curve (see Fig. 4A), and unlike the change in gain is consistent with an additive process [33].

### Noise normalises gain

**Figure 5:**
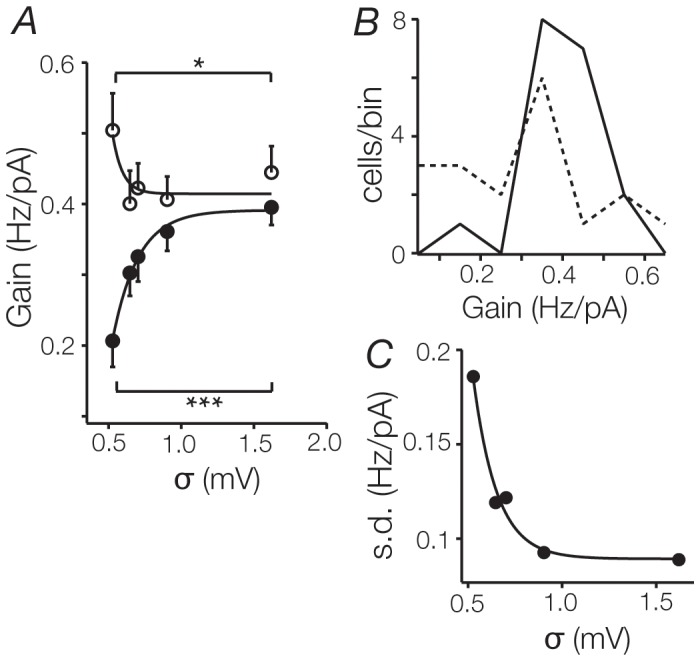
Noise normalises gain changes. **A**. Gain changes were not uniform within the recorded population, as noise reduced gain in cells with initially high gains (n  =  5, open circles), and increased gain in those with low initial gains (n  =  13, closed circles). **B.** Histograms of gains across the sample population (n  =  18) under control conditions (dashed line) and for the highest level of noise (σ_50_, solid line). Note the sharper distribution of gains under noisy conditions. **C**. The standard deviation of the average of gains across the population, plotted against the corresponding noise level. The standard deviation is reduced by 52% at high noise levels. Data were fit with an inverse exponential function.

Although the addition of noise on average increased the gain of TC cells, in 5 of 18 cells tested the gain significantly decreased (for this sample, σ_0_: 0.50 ± 0.05 Hz/pA, σ_50_: 0.44 ± 0.03 Hz/pA; n  =  5, p  =  0.02). Figure 5A plots gain as a function of noise amplitude for those cells where gain increased (solid circles) and those where it decreased (open circles). The impact of the noise depends on the initial gain of the cell, so that those with high gain in the absence of noise are attenuated by noise and vice versa. To further characterise this, we plotted histograms for the distribution of gains under both control and noisy conditions (Figure 5B). Under noisy conditions, the range of gains was 34% smaller than under control conditions (noise: 0.20–0.55 Hz/pA, control: 0.0–0.68 Hz/pA), suggesting a narrowing of the distribution. The standard deviation (SD) of the distribution in the presence of noise was correspondingly reduced by 52% when compared to measurements obtained without noise (noise: 0.08 Hz/pA, control: 0.19 Hz/pA; Fig. 5C). These changes indicate that noise decreases the variability in sensitivity between cells. This noise-induced reduction in variability was also evident in the thresholds: the range decreased by 30% (noise: 70 pA, control: 100 pA), and the SD of the distribution decreased by 39% (noise: 22 pA, control: 36 pA). No other recorded parameters distinguished cells with high and low gain.

### Postsynaptic current noise can induce gain changes

**Figure 6:**
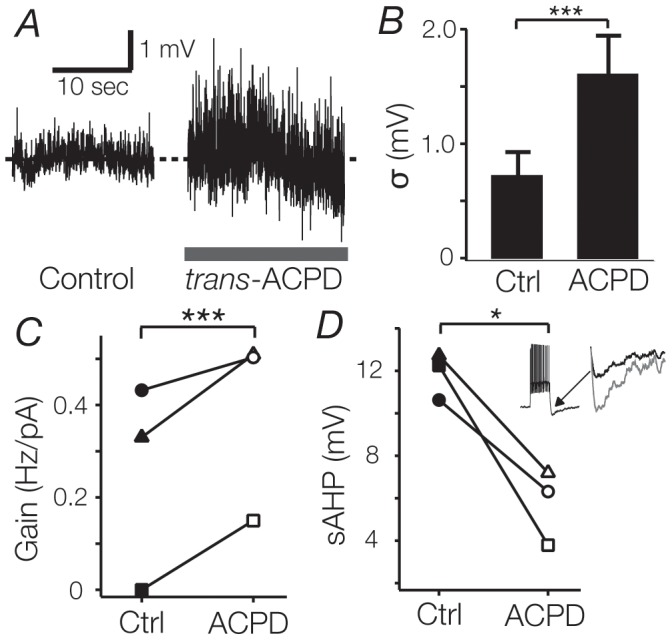
Increased synaptic noise induces multiplicative gain changes. **A and B**. Bath-application of 250 µM trans-ACPD increased the standard deviation of the membrane potential in TC cells. Note the large increase in activity centred around baseline (dashed line, -67 mV) in A. The increase in SD is comparable to the highest level of current noise (1.57 vs 1.6 ). **C**. For each individual cell (n  =  3) bath application of trans-ACPD increased gain in comparison to control. The increase in gain was paralleled by a reduction in the amplitude of the sAHP (**D**; main panel and inset).

Excitatory corticothalamic synapses, mediated by postsynaptic mGluR1a glutamate receptors [11], account for about 30% of the total synaptic input onto TC cells in dLGN [20], [34]. These corticothalamic synapses may act as a potent source of synaptic noise, and thereby modulate the input sensitivity of TC cells. To test this hypothesis, we bath-applied the mGluR1a agonist trans-ACPD [35]. In 7 cells tested, bath-application of trans-ACPD significantly depolarised the membrane (trans-ACPD: -62.9 ± 4.5 mV; control: -66.8 ± 4.6 mV; n  =  7, p < 0.005), increased the standard deviation of the membrane potential (trans-ACPD: 1.6 ± 0.34 mV; control: 0.72 ± 0.21 mV; n  =  7, p < 0.01; Figures 6A & B). The SD of the membrane potential during trans-ACPD application approximated that produced by the highest level of current noise (1.57 mV). To determine whether pharmacologically induced synaptic activity would induce gain changes similar to those seen with noisy current stimuli, we delivered noiseless current pulses to 3 cells during the application of trans-ACPD. Gain significantly increased in each of these cells (trans-ACPD: 0.39 ± 0.12 Hz/pA; control: 0.25 ± 0.13 Hz/pA; n  =  3, p  =  0.03, Fig. 6C), while threshold tended to decrease (trans-ACPD: 187 ± 87 pA; control: 293 ± 59 pA; n  =  3, p  =  0.11), along with the input resistance (trans-ACPD: 27 MΩ; control: 53 MΩ; n  =  3, p  =  0.11). Unlike the case with simulated current noise, trans-ACPD always produced an increase in gain in the recorded cell, regardless of the initial gain.

### Synaptic noise, but not current noise, changes sAHP

Previous work on somatosensory pyramidal cells has shown that blockade of currents that mediate the stimulus afterhyperpolarisation (sAHP, see inset Figure 6D) lead to a decrease in input sensitivity [26]. This suggests that in the normal state these currents contribute significantly to the capacity of neurons to sustain high input sensitivity. However, the increase in input sensitivity seen during bath-application of trans-ACPD was correlated with a decrease in sAHP amplitude. Application of trans-ACPD reduced the sAHP produced by noiseless current steps (trans-ACPD: 5.8 mV; control: 12 mV; n  =  3; p  =  0.02), while current noise did not lead to a change in the sAHP, regardless of the noise amplitude (σ_0_: 6.8 mV; σ_50_: 6.7 mV; n  =  18; p  =  0.39; Figure 6D). Given that high gains were sustained in the absence of a significant sAHP current contribution, these results suggest that this component may be sufficient, but not necessary for the expression of high input sensitivity in all neurons.

### T-type channel blockade induces gain changes

**Figure 7:**
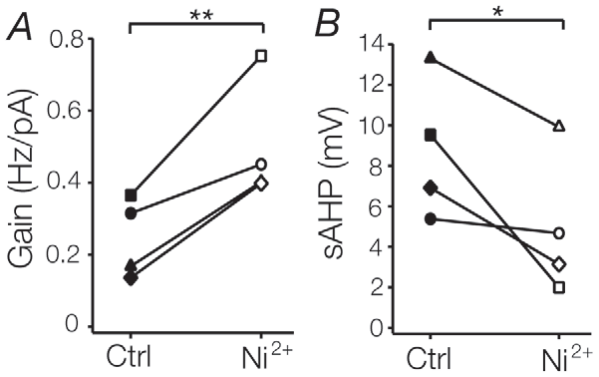
T-type Ca^2+^ channel block induces additive and multiplicative gain changes. **A**. Graph plotting the increase in gain during bath-application of 250 µM Ni^2+^ for each recorded cell. **B**. As in **A**., with sAHP plotted for each cell.

Evidently, changing the spectrum of currents active during the cell’s response can induce significant gain changes. To see which components may be necessary for such changes, we used Ni^2+^ to block T-type Ca^2+^ channel mediated currents. In all 4 cells tested, bath application of 250 µM Ni^2+^ significantly increased gain (Ni^2+^: 0.50 ± 0.08 Hz/pA, control: 0.25 ± 0.05 Hz/pA, p  =  0.01; Figure 7A) and decreased threshold (Ni^2+^: 150 ± 25 pA, control: 245 ± 41 pA; p  =  0.03). Interestingly, application of Ni^2+^ also reduced the sAHP produced by noiseless current steps (Ni^2+^: 3.1 mV; control: 6.9 mV; n  =  4, p  =  0.03; Figure 7B). Membrane potential (Ni^2+^: -69.8 mV; control: -67.9 mV; n  =  4, p  =  0.07) and input resistance (Ni^2+^: 81 MΩ; control: 102 MΩ; n  =  4, p  =  0.1) did not change significantly with the application of nickel. These results suggest that T-type channels may serve to dampen sensitivity in TC neurons not only by increasing the threshold from which tonic action potentials can be fired (the additive component), but also by limiting firing frequency at much higher membrane potentials.

## Discussion

Our experiments demonstrate that TC neurons display a range of sensitivities (based on threshold and gain measurements), which are significantly modified by the injection of current noise. This modification is dependent upon the intrinsic sensitivity of each neuron, such that those with high gain in the absence of noise are attenuated by noise and vice versa, suggesting that noise normalises the gain of TC neurons to an optimal value. Changes in intrinsic sensitivity are also induced by pharmacological agents that either activate specific modulatory postsynaptic receptors (trans-ACPD) or block active currents (Ni^2+^), further suggesting that the normalisation of gains may reflect the prevailing level of synaptic input onto neurons and adjust their sensitivity accordingly.

In our experiments, the introduction of small amounts of current noise caused significant changes in gain that were dependent upon the initial intrinsic neuronal sensitivities (e.g. gain was reduced in high sensitivity cells). We chose to restrict the maximum injected current noise, by setting a level of membrane potential fluctuation similar to the level induced by the bath-application of the metabotropic glutamate receptor agonist trans-ACPD. As such, our results reliably reproduce values of membrane potential fluctuation that are physiologically relevant. Despite this, our results are in contrast to the findings of Chance et al. (2002) and Wolfart et al. (2005), whose studies demonstrate only divisive changes (reductions) in gain following the injection of current noise into rat somatosensory cortical and guinea pig thalamic neurons respectively. However, more recent work by Higgs et al. (2006) has demonstrated that current noise increases gain in pyramidal neurons, but decreases gain in fast-spiking interneurons. Taken together, these studies suggest that noise may alter neuronal sensitivity in a neuron-specific manner, and our own results are concordant with this hypothesis. Further, the bidirectional changes in gain in response to sub-threshold noise may be specific to cells that exist in bi-stable states. Therefore, gain changes observed in the response of different types of neuron may reflect specific firing characteristics. For example, type B vestibular cells [36], spinal motoneurons [37] and olfactory bulb mitral cells [38] have been shown to exist in bistable states and may also show normalization of gain functions. In addition other cells that display non-classical, non-linear firing response characteristics (eg. cerebellar Purkinje neurons, intrinsically bursting neocortical neurons) may also utilize this type of operation when scaling their output.

Previous work on pyramidal cells in mouse somatosensory cortex [26] has shown that input sensitivity is partly governed by the sAHP. Specifically, addition of the 5-HT_2_ agonist α-methyl-5-HT to block sAHP currents lead to an increase in the additive component and a decrease in the multiplicative component, suggesting that in the normal state these currents contribute significantly to the capacity of neurons to sustain high input sensitivity. In contrast, our results show that, at least in TC neurons, a reduction of sAHP current amplitude is correlated with increasing sensitivity, implying that there may be other currents that help modulate sensitivity in these cells. For example, the potassium currents mediated by Kv3 channels [39] and/or the slow conductance (SK) calcium-dependent potassium channels, which have been suggested to link functionally with T-type calcium channels in muscle cells and neurons [40], [41], [42]. These results suggest that there may be a significant redundancy in the mechanisms that govern this process. This is not surprising, considering the heterogeneity of ion channel profiles displayed across neurons. However, from our experiments it is not possible to determine whether this changing current contributes to the mechanism that maintains high input sensitivities, or whether it represents an epiphenomenon.

### Impact of pharmacological stimuli on sensitivity

Pharmacological experiments have the advantage over current injections of being able to mimic *in vivo* situations more accurately. Our experiments show that the sensitivity of TC cells can be modified by selective pharmacological stimulation or blockade of ion channels and/or receptors. Previous experiments have demonstrated that excitatory postsynaptic inputs mediated by metabotropic glutamate receptors (mGluR1a) may selectively enhance neuronal sensitivity to particular visual inputs [12], [43], [44], [45]. Our results are consistent with this finding, showing that background excitatory receptor activation can increase the input sensitivity of TC neurons (see figures 4 and 6). In addition, our experiments showed that pharmacological blockade of T-type calcium channels with nickel causes a significant decrease in T-type currents. While nickel is a widely used blocker for T-type channels it is not generally considered the most selective agent for this purpose, indeed nickel has also been shown to block L-type calcium channels at higher doses [46]. Novel, reversible T-type channel antagonists (3,5-dichloro-N-[1-(2,2-dimethyl-tetrahydro-pyran-4-ylmethyl)-4-fluoro-piperidin-4-ylmethyl]-benzamide, also known as TTA-P2) with much greater efficacy than nickel have recently been identified [47] and may provide more specific information regarding the role of T-type channels in controlling neuronal sensitivity. It is unlikely however, that the pattern of sensitivity changes reported here will differ significantly using an alternative antagonist. While our results demonstrate that TC neuron sensitivity is modified by trans-ACPD and nickel, we presume that other compounds and their associated targets also contribute. For example in the mouse vestibular nucleus glycine receptor mutations result in chronic alterations in type B vestibular nucleus neuron sensitivity [27]. As such we expect that inhibitory inputs (most likely GABAergic) may also contribute to TC neuron sensitivity [43], [44].

### Functional role of sensitivity normalization

Since TC cells fire in burst and tonic mode, the normalization function of current noise may operate to maintain this bi-stability. There are two possible roles this operation may have, each working on different timescales. 1) A normalisation operation may set the mean response range for cells in particular sensory situations, which may play an important role in synchronising outputs of groups of cells. 2) It may play a protective role by preventing neuronal excitotoxicity and network over-excitation. In the former, TC cells receiving convergent inputs from the periphery (e.g. the retina) are able to adjust their output to the prevailing level of stimulation on the millisecond timescale. For example, in the case of strong retinal stimulation the neuronal sensitivity of the dLGN is reduced to preserve the contrast sensitivity of the visual pathway- in much the same way as contrast adaptation in M-pathway retinal ganglion cells [48]. In the latter, neurons chronically deprived of inhibitory control can reduce the expression of voltage sensitive channels and thus avoid the damaging effects of over-excitation (E.g. calcium excitotoxicity). For example, mouse MVN neurons chronically deprived of glycinergic inhibition display significantly reduced gain in response to current injection *in vitro* [27]. Regardless of the timescale, it seems reasonable to conclude that the output of individual neurons is ultimately the result of short and long-term adaptations to network activation levels.
